# Cerebral volumes, neuronal integrity and brain inflammation measured by MRI in patients receiving PI monotherapy or triple therapy

**DOI:** 10.7448/IAS.17.4.19578

**Published:** 2014-11-02

**Authors:** Ignacio Pérez Valero, Alicia Gonzalez Baeza, Juan Antonio Hernandez-Tamames, Susana Monge, Francisco Arnalich, Jose Ramon Arribas

**Affiliations:** 1 Internal Medicine, H.U. La Paz, Madrid, Spain; 2 Electronic Department, Universidad Rey Juan Carlos, Madrid, Spain; 3 Instituto de Salud Carlos III, Centro Nacional de Epidemiología, Madrid, Spain

## Abstract

**Introduction:**

Penetration of protease inhibitors (PI) in the central nervous system (CNS) is limited. Therefore, there are concerns about the capacity of PI monotherapy (MT) to control HIV in CNS and preserve brain integrity.

**Methods:**

Exploratory case-control study designed to compare neuronal integrity and brain inflammation in HIV-suppressed patients (>2 years) with and without neurocognitive impairment (NI), treated with MT or triple therapy (TT), 3-Tesla cerebral magnetic resonance image (MRI) and spectroscopy (MRS) were used to evaluate neuronal integrity (volume of cerebral structures and MRS levels of N-acetyl-aspartate (NAA)) and brain inflammation (MRS levels of myo-inositol (MI) and choline (CHO)). MRS biomarkers were measured in 4 voxels located in basal ganglia, frontal (2) and parietal lobes. A comprehensive battery of tests (14 tests - 7 domains) was used to diagnose neurocognitive impairment [1].

**Results:**

We included 18 neurocognitively impaired patients (MT: 10, TT: 8) and 21 without NI (MT: 9; TT: 12, Table 1). Subset of patients with NI: cerebral volumes and MRS biomarkers were mostly similar between MT and TT with exception of the right cingulate nucleolus volume (MT: 8854±1851 vs TT: 10482±1107 mm^3^; p<0.04), CHO levels in basal ganglia (MT: 0.44±0.05 vs TT: 0.37±0.03 MMOL/L; p<0.01) and the NAA levels in parietal lobe (MT: 1.49±0.12 vs 1.70±0.13 MMOL/L; p<0.01). Subset of patients without NI: cerebral volumes and MRS biomarkers were mostly similar between MT and TT with exception of MI levels in frontal lobe (MT: 1.20±0.36 vs 0.81±0.25 MMOL/L; p=0.01).

**Conclusions:**

We did not find significant differences in cerebral volumes or MRS biomarkers in most areas of the brain. However, we found higher levels of inflammation and neuronal damage in some brain areas of patients who received MT. This observation has to be taken into caution while we could not adjust our results by potential confounders. Further investigation is needed to confirm these preliminary results.

**Table 1:** Main baseline characteristics by presence of neurocognitive impairment and type of antiretroviral therapy

	Impaired cognitive functioning			Normal cognitive functioning		
				
	Monotherapy n=10	Triple therapy n=8	p value	Monotherapy n=9	Triple therapy n=12	p value
Age, mean (DS)	49.5±8.6	40.1±8.0	0.03	48.6±9.2	46.6±3.4	0.49
Gender: male, n (%)	5 (50)	5 (62.5)	0.60	7 (77.8)	8 (66.7)	0.57
HCV antibody +, n (%)	3 (30)	2 (25)	0.53	4 (44.4)	4 (33.3)	0.67
CD4 nadir, median (IQR)	210 (71–323)	73.5 (14–221)	0.17	204 (187–309)	178 (59–284)	0.08
Time since HIV+, median (IQR)	15.6 (12.9–17.9)	9.9 (4.6–20.5)	0.18	16 (13.2–22.3)	14.9 (8.6–20.9)	0.45
Time of HIV-suppression, median (IQR)	9.1 (6.3–11.2)	3.4 (3–5.6)	0.01	5.5 (4.4–11)	7.1 (4.6–11.4)	0.73
Time on antiretroviral therapy, median (IQR)	13.2 (12–15)	5.9 (3.8–13.9)	0.01	12.7 (7.2–16.7)	10.3 (6.3–16.5)	0.73
